# Tailored birdcage resonator for magnetic resonance imaging at 7 T using 3D printing

**DOI:** 10.1016/j.ohx.2022.e00326

**Published:** 2022-06-09

**Authors:** Philip Kemper, Jorg Thöming, Ekkehard Küstermann

**Affiliations:** a Chemical Process Engineering (CVT), Faculty of Production Engineering (FB 4), University of Bremen, Leobener Straße 6, 28359 Bremen, Germany; b In vivo MR Group, Faculty of Chemistry (FB 2), University of Bremen, Leobener Straße NW2, 28359 Bremen, Germany

**Keywords:** Magnetic resonance imaging, Birdcage resonator, RF coil, 3D printing, Medical hardware

## Abstract

This study outlines the design, construction and characterization of a tailored low-cost linear birdcage (BC) resonator for magnetic resonance imaging at 7T. Typically, different BC designs found in literature are well described in theory but lack crucial information for practical realization. This is challenging, as theoretical and practical aspects often differ greatly from each other, especially in the field of high frequency technology. We propose a simple and open-source 3D printable design which results in a working BC if the instructions in this publication are followed. The aim is to open up the possibility of building a functioning BC with simple means and a budget below 750 €, even for users without a great deal of expertise in MRI coil building. We demonstrate that the BC can achieve a good $B_{1}$ field homogeneity using the double angle method. The proposed design is qualitatively compared to a commercially available resonator. Both perform equally well in terms of SNR and image quality.

## Graphical abstract

**Figure:**
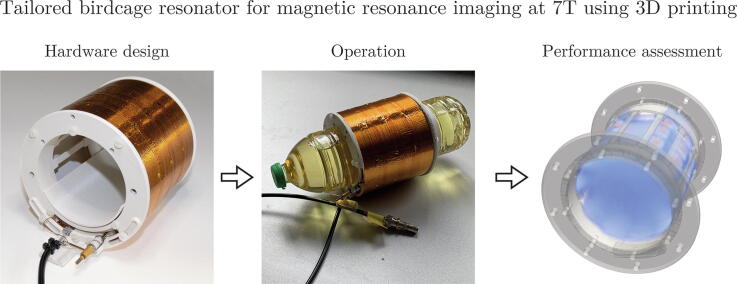

**Specifications table.**

**Table:** 

**Hardware name**	Inductively coupled linear birdcage resonator for MRI at 7T	
**Subject area**	• Magnetic resonance imaging • Chemical engineering • Biomedical engineering	
**Hardware type**	• Imaging tools	
**Closest commercial analog**	”No commercial analog is available.”	
**Open source license**	Creative Commons Attribution-ShareAlike 4.0 International License (CC BY-SA 4.0)	
**Cost of hardware**	750 €	
**Source file repository**	https://doi.org/10.17605/OSF.IO/29A8T	

## 1 Hardware in context

Magnetic resonance imaging (MRI) is a non-invasive visualization technique widely used in medical diagnostics and other fields of research such as chemical engineering. Key component of the MRI system is the radio frequency (RF) coil used for sending an oscillating magnetic field ($B_{1}$) which interacts with the specimen inside the static magnetic field ($B_{0}$) of the MR scanner and for receiving the electromagnetic response of the sample to the previously applied $B_{1}$ field.

A large number of different surface and volume coil variants can be found in literature [1], [2] as no universally applicable design exists. Commercially available coils are generally limited to a specific use case and thus often restricted in terms of size and sensitivity. To ensure optimum measurement results, the RF coils must be tailored to the respective problem. The use of birdcage (BC) resonators is most common for volumetric samples with diameters lager than 10mm. [3].

The BC resonator was first introduced in 1985 by Hayes et al. [4] for head and body imaging and has since then been adopted to a wide range of applications [5], [6], [7], [8], [9]. A clear advantage over a saddle coil or a slotted tube resonator was the significantly increased RF field homogeneity and a better signal to noise (SNR) ratio. The common BC is a cylindrical volume type RF resonator consisting of two end rings connected by a usually even number of equally spaced conductor segments referred to as legs or rungs. The BC works on the principle of a resonant *LC* circuit, with the end rings and the conductor segments representing the inductive (*L*) part. The capacitive (*C*) part is determined by lumped capacitors soldered to the resonator. Three different types of BC resonators can be distinguished, depending on the placement of the capacitors. In a low pass (LP) configuration, the capacitors are placed onto the legs of the BC, whereas they are distributed on the end rings when using a high pass (HP) configuration. In band pass (BP) mode, capacitors are located on both, legs and end rings. Often the LP mode is preferred, as it uses the least number of components. [10].

Resonance frequencies of all three configurations can be determined using analytical or numerical methods [11], [12], [13], [14]. However, these estimates usually do not account for the actual behaviour of the resonator when it is connected to the MRI console or for possible influences of shielding and dielectric properties of materials used in construction. Considerable deviations can occur in practice, especially when designing resonators for ultra-high field ($B_{0}$ ⩾7T) applications. As a result, the seemingly simple to realize BC design will not work in the desired frequency range or cannot be tuned and matched at all. Often, the published BC designs lack essential information on the design decisions made, which makes it impossible for non-experts to recreate a working coil.

The design, construction and characterization of a tailored low-cost linear BC resonator for magnetic resonance imaging at $B_{0}$ = 7T was performed in this study. The proposed open-source 3D printable design was developed in order to help users without a major experience in MRI coil building to assemble a fully working resonator by following the here given instructions. The costs were kept below 1000 € as only a limited number of components was needed. Thoughts and motivations behind the taken design decisions were disclosed for a better understanding. The homogeneity of the $B_{1}$ field was demonstrated using the double angle method [15]. A comparison to a commercially available resonator was conducted, at which both resonators performed equally well in terms of SNR and image quality.

## 2 Hardware description

The proposed design offers a low-cost and open-source alternative to commercially available birdcage resonators. All structural parts are 3D printable and other components are easy to obtain. The first step in designing a BC resonator is the determination of the required geometric parameters and operating conditions.

Experiments were performed inside a 7T NMR imaging system (Biospec 70/20, Bruker Biospin GmbH, Ettlingen, Germany) equipped with a BGA12S2 gradient system (441mTm^−1^ maximum gradient strength), with a working frequency of $f_{0}$ = 300.3MHz and a bore diameter of $d_{\mathrm{bore}}$ = 116mm. A shield was preferred to minimize potential effects from external interference.

**Fig. 1:**
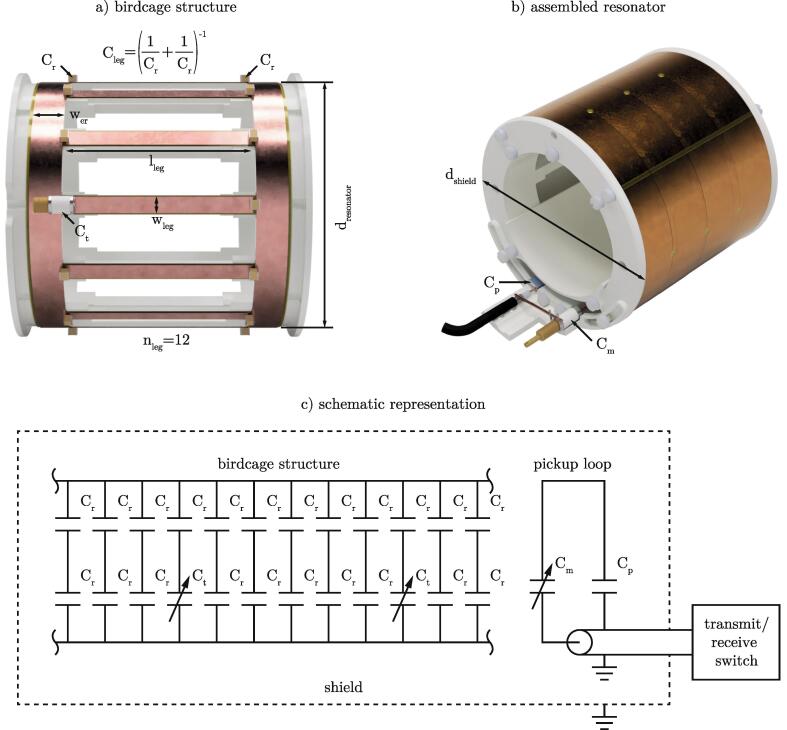
Main variables of the hardware a, b) and schematic representation of the BC resonator c).

The Python3 version *pyBirdcagebuilder* [16] of the original web-based java application *BirdcageBuilder* [17] was used to calculate preliminary design and component values (see Table 1) based on the aforementioned factors. A detailed overview of the software is given by Chin et al. [11]. The main variables of the hardware listed in Table 1 are illustrated in Fig. 1a, b).

**Table 1:** Preliminary design and component values obtained from *pyBirdcagebuilder*.

	**calculation**	**construction**
configuration	low pass	low pass
resonant frequency $f_{0}$	300MHz	300MHz
type of leg and endring	rectangular	rectangular
number of legs $n_{\mathrm{leg}}$	12	12
coil diameter $d_{\mathrm{resonator}}$	83mm	83mm
leg length $l_{\mathrm{leg}}$	60mm	58mm
leg width $w_{\mathrm{leg}}$	5mm	5mm
endring segment width $w_{\mathrm{er}}$	10mm	10mm
RF shield diameter $d_{\mathrm{shield}}$	110mm	110mm
capacitance $C_{\mathrm{leg}}$	2.03pF	2.35pF (4.7pF + 4.7pF)

The presented setup was designed aiming at a maximum inner diameter for large samples to perform hydrodynamic experiments of gas–liquid flows in vertical glass capillaries, similar to the work presented by Kemper et al. [18]. As the sample length was not an issue, the BC was kept short. According to Mispelter et al. [10] the maximum amplitude of the magnetic field at the coil’s center is reached at a length of $l=d\cdot \sqrt{2}$ whereas optimum homogeneity is obtained at l=d·1.661. They also pointed out an important aspect regarding the ratio between shield and resonator diameter $d_{\mathrm{shield}}$/$d_{\mathrm{resonator}}$ that should be taken into account during the design process. With a decreasing diameter ratio, the magnetic field concentrates in the region between the shield and the resonator, resulting in a significant decrease of the magnetic field amplitude inside the resonator. No specific suggestions were given, but the ratio should be as large as possible to avoid the previously mentioned effect. Here, a ratio of $d_{\mathrm{shield}}$/$d_{\mathrm{resonator}}$ = 1.2 was chosen. It is possible to tune the resonant frequency of the shield in case of interference with the resonant frequency of the NMR. For this purpose, a slotted shield design with additional capacitors for frequency matching is often utilized. Here, a fully closed cylindrical copper surface was used as no interference with the NMR’s resonant frequency was observed. Conductive copper adhesive tape is particularly suitable for this purpose, as it can be easily attached to the cylindrical body of the shield.

A twelve leg low pass design was picked as it offered a good compromise between constructional complexity and image quality. With increasing number of legs, the homogeneity of the magnetic field improves. At the same time, the sensitivity to current errors is reduced [10]. The schematic representation of the BC is shown in Fig. 1c). Instead of using a single capacitor in the center of each leg with the calculated value of $C_{\mathrm{leg}}$ = 2.03pF, the capacitance was split into two identical capacitors with approximately double the value of $C_{\mathrm{leg}}$. With two capacitors of $C_{r}$ = 4.7pF connected in series, a total value of $C_{\mathrm{leg}}$ = 2.35pF was archived. Placing them on both ends of each leg had two advantages. Firstly, possible field disturbances at the capacitors were moved away from the region of interest (ROI), namely the resonator’s center. Secondly, the design was simplified, as the capacitors provided the connection between the leg and the end ring.Two of the fixed value capacitors $C_{r}$ were replaced by two variable trimmers (NMKP4HV, Knowles) $C_{t}$ = 0.6pF–4.0pF on opposite legs for frequency tuning. The use of two diametrically opposed trimmers helps to adjust the current distribution and thus maintain the field symmetry of the resonator.

When interfacing the BC to the transmit and receive switch of the MRI system, the energy transfer between the probe and the transceiver needs to be optimized for best results. For this, it is important to match the impedance of the resonator to a purely resistive impedance of 50Ω [2]. Inductive coupling was implemented to interface and match the BC to the transmit and receive switch. Matching is achieved by either altering the distance between the pickup loop and the resonator or by adjusting the capacitance of the pickup loop. The latter was done here, as the distance was fixed by design. A tunable rectangular pickup loop was constructed from copper wire ($d_{\mathrm{wire}}$ = 1.7mm) with a fixed value capacitor $C_{p}$ = 4.7pF (Knowles) connected in series to a variable capacitor (NMAP40HVFS, Knowles) $C_{m}$ = 1.5pF–40pF for impedance matching. The value of $C_{p}$ was determined by trial and error, testing different values between 1.0pF–47pF (Knowles). The pickup loop was aligned parallel to the resonator in between the birdcage structure and the shield. Both ends can be rotated to a certain extend for precise parallel alignment of the loop. For practical reasons, the loop can be positioned parallel to a leg or the space in between two rungs. The final polarization of the magnetic field is defined by the position of the pickup loop inside the MR scanner, as the $B_{1}$ field moves perpendicular to the loop [10]. The pickup loop position and thus the magnetic field polarization may be arbitrarily chosen, depending on the experiment. Capacitive coupling of the BC is also possible, but usually more difficult to implement than the inductive approach. The tune and match network needs to be precisely calculated or measured. It is soldered directly to the coil, introducing a load which renders a perfect current distribution between the legs almost impossible, thus reducing performance [10].

The resonant frequency $f_{0}$ of an *LC* circuit is calculated as follows: $f_{0}=1/(2\pi \sqrt{\mathrm{LC}})$. In case the frequency of the resonator is outside the desired range, it can be fixed by adjusting the capacitance or the inductance of the BC. The easiest way is to replace the capacitors, provided that suitable values are available. The resonant frequency decreases with increasing values of *C*. Alternatively, the inductance *L* of the legs can be increased by making them longer, resulting in a smaller resonant frequency $f_{0}$.

In a similar approach as explained before, resonators can be designed for different problems and experiments. The range of applications for the described hardware is vast and versatile. For example, a BC could be tailored specifically to study extracted rodent brains. The resonator’s diameter could therefore be reduced and the number of rungs increased to obtain a higher SNR. One could also imagine the construction of a sufficiently long BC with moderate diameter to investigate flow and mixing phenomena in horizontal pipe flow of liquid–liquid systems, which are of particular interest in the oil industry. Further applications in optically inaccessible systems are imaginable. For example, the root growth of plants in soil, the catalytic reaction of chemicals in opaque monolithic reactors or the diffusion of substances through different media can be studied. Each application places unique requirements on the measurement hardware which can be taken into account during the design and construction of a BC resonator.

## 3 Design files summary

A summary of all design files is listed in Table 2. 3D printed parts are labeled with the abbreviation FFF (fused filament fabrication). The function of the parts is given by the designation. All files are available in the online repository: https://doi.org/10.17605/OSF.IO/29A8T

**Table 2:** Summary of all design files.

**Design file name**	**File type**	**Open source license**
FFF_endring_trimmer	Fusion 360 file (*.f3d), STL file	CC BY-SA 4.0
FFF_endring_no_trimmer	Fusion 360 file (*.f3d), STL file	CC BY-SA 4.0
FFF_rung	Fusion 360 file (*.f3d), STL file	CC BY-SA 4.0
FFF_endplate_trimmer	Fusion 360 file (*.f3d), STL file	CC BY-SA 4.0
FFF_endplate_no_trimmer	Fusion 360 file (*.f3d), STL file	CC BY-SA 4.0
FFF_holder_pickup_loop	Fusion 360 file (*.f3d), STL file	CC BY-SA 4.0
FFF_endplate_pickup_loop_trimmer	Fusion 360 file (*.f3d), STL file	CC BY-SA 4.0
FFF_endplate_pickup_loop_no_trimmer	Fusion 360 file (*.f3d), STL file	CC BY-SA 4.0
FFF_shield	Fusion 360 file (*.f3d), STL file	CC BY-SA 4.0
FFF_sample_tray	Fusion 360 file (*.f3d), STL file	CC BY-SA 4.0
Model_birdcage	Fusion 360 file (*.iges)	CC BY-SA 4.0

## 4 Bill of materials

The complete bill of material (BOM) with the necessary parts to rebuild the resonator is listed in Table 3. The listed components may also be obtained from different suppliers.

**Table 3:** Summary of all components.

**Designator/ name**	**Number**	**Cost/unit**	**Total cost**	**Source of Material**	**Material type**
FFF_endring_trimmer	1	21.81 €/kg	0.35 €	Das Filament	PLA
FFF_endring_no_trimmer	1	21.81 €/kg	0.35 €	Das Filament	PLA
FFF_rung	12	21.81 €/kg	0.26 €	Das Filament	PLA
FFF_endplate_trimmer	1	21.81 €/kg	0.39 €	Das Filament	PLA
FFF_endplate_no_trimmer	1	21.81 €/kg	0.37 €	Das Filament	PLA
FFF_holder_pickup_loop	2	21.81 €/kg	0.17 €	Das Filament	PLA
FFF_endplate_pickup_loop_trimmer	1	21.81 €/kg	0.11 €	Das Filament	PLA
FFF_endplate_pickup_loop_no_trimmer	1	21.81 €/kg	0.04 €	Das Filament	PLA
FFF_shield	1	21.81 €/kg	1.37 €	Das Filament	PLA
FFF_sample_tray	1	21.81 €/kg	0.37 €	Das Filament	PLA
Slotted screw M4 x 8mm					
(Essentra - 50M040070K008)	18	0.16 €	2.88 €	Mouser	Nylon
Slotted screw M4 x 10mm					
(Essentra - 50M040070K010)	6	0.16 €	0.96 €	Mouser	Nylon
Self adhesive Copper tape 25.4mm					
(3 M - 1181 TAPE 1”)	1	66.42 €/roll	66.42 €	Mouser	Copper
Kapton tape 6.35mm					
(3 M - 1205–1/4”)	1	18.41 €/roll	18.41 €	Mouser	Polyimide
Kapton tape 25.4mm					
(3 M - 1205–1”)	1	59.37 €/roll	59.37 €	Mouser	Polyimide
Enameled copper wire, solid core, AWG 24, $d_{\mathrm{wire}}$ = 0.51mm					
(Remington Ind. - 24SNSP.125)	1	11.17 €	11.17 €	Digikey	Copper
Insulated copper wire, solid core, AWG 14, $d_{\mathrm{wire}}$ = 1.63mm					
(Alpha Wire - 541401 RD005)	1	45.63 €	45.63 €	Mouser	Copper
Variable capacitor 0.6pF–4.0pF					
(Knowles - NMKP4HV)	2	134.90 €	269.80 €	Mouser	Semiconductor
Variable capacitor 1.5pF–40pF					
(Knowles - NMAP40HVE)	1	167.54 €	167.54 €	Mouser	Semiconductor
Ceramic capacitor 4.7pF, 2kV, non-magnetic, high Q (Knowles - 111122K04P70BQTAF9LM)	22	1.56 €	34.32 €	Digikey	Semiconductor
Ceramic capacitor 4.7pF, 3.6kV, non-magnetic, high Q (Knowles - 222523K64P70BQTAF9LM)	1	4.96 €	4.96 €	Digikey	Semiconductor
BNC cable, male/male, 50Ω					
(Amphenol - 095–850-277M050)	1	18.24 €	18.24 €	Mouser	Non-specific
Cable ties					
(Adv. Cable Ties - AL-14–40-0-C)	1	9.91 €/pack	9.91 €	Digikey	Nylon
Super glue gel, fast curing					
(Surehold - 78-SH-376)	1	1.19 €	1.19 €	Digikey	Cyanoacrylate
**Total cost**			**714.58** €		

## 5 Build instructions

**Fig. 2:**
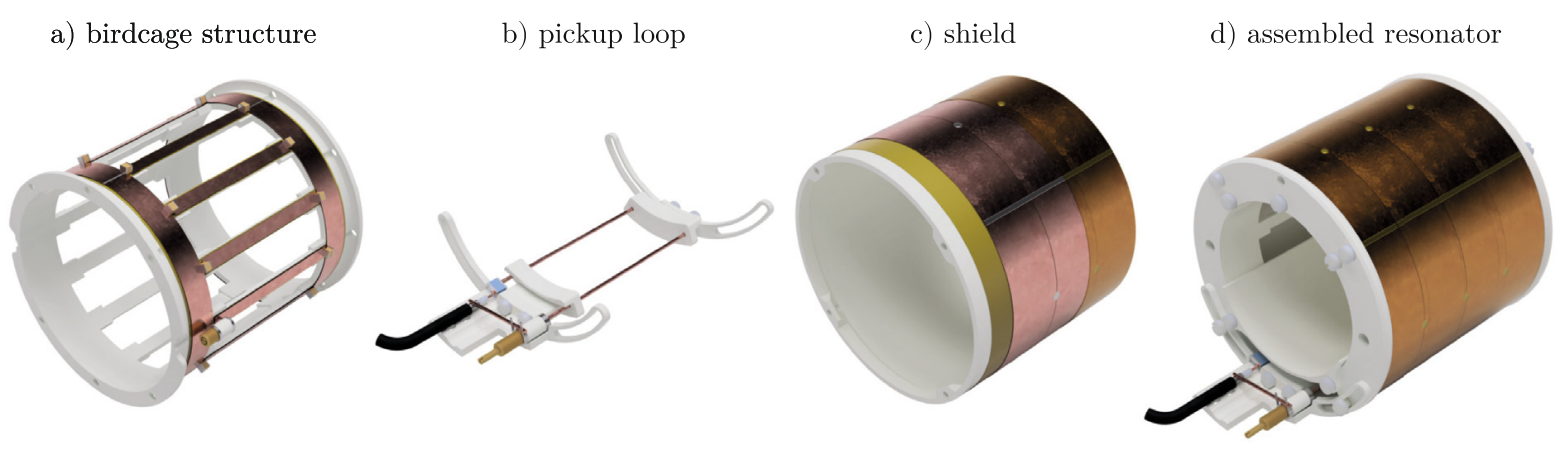
Components of the BC resonator. The resonant structure a) consists of lumped elements soldered onto copper stripes mounted on a 3D-printed support structure. A bent copper wire and two capacitors form the pickup loop b). A cut through the multiple layers of the shield is depicted in c). The components a-c) are combined to the assembled resonator d).

The entire BC structure was produced using fused filament fabrication 3D printing. The corresponding parts are listed in Table 2 and are indicated with FFF in the name. All other components were purchased online (BOM, Table 3). The parts were printed from PLA with 0.2mm layer height, 4/4/4 top/bottom/wall layers and 25% cubic infill. All parts can be printed without supports. Depending on the 3D printer used, surface roughness and imperfections may occur and should be removed with the help of a cutter knife or sandpaper for best fitting accuracy. An overview of the different constructed components is given in Fig. 2.

The following additional tools are required for construction and assembly of the resonator:

1. Soldering iron and lead-free solder
2. Hot glue gun
3. Pliers
4. Side cutter
5. Cutter knife
6. Tweezers
7. Flathead screwdriver
8. Ruler
9. Small spring clamps
10. Fine sandpaper
11. M4 tap
12. Isopropanol
13. Multimeter

### 5.1 Assembly of the BC structure

**Fig. 3:**
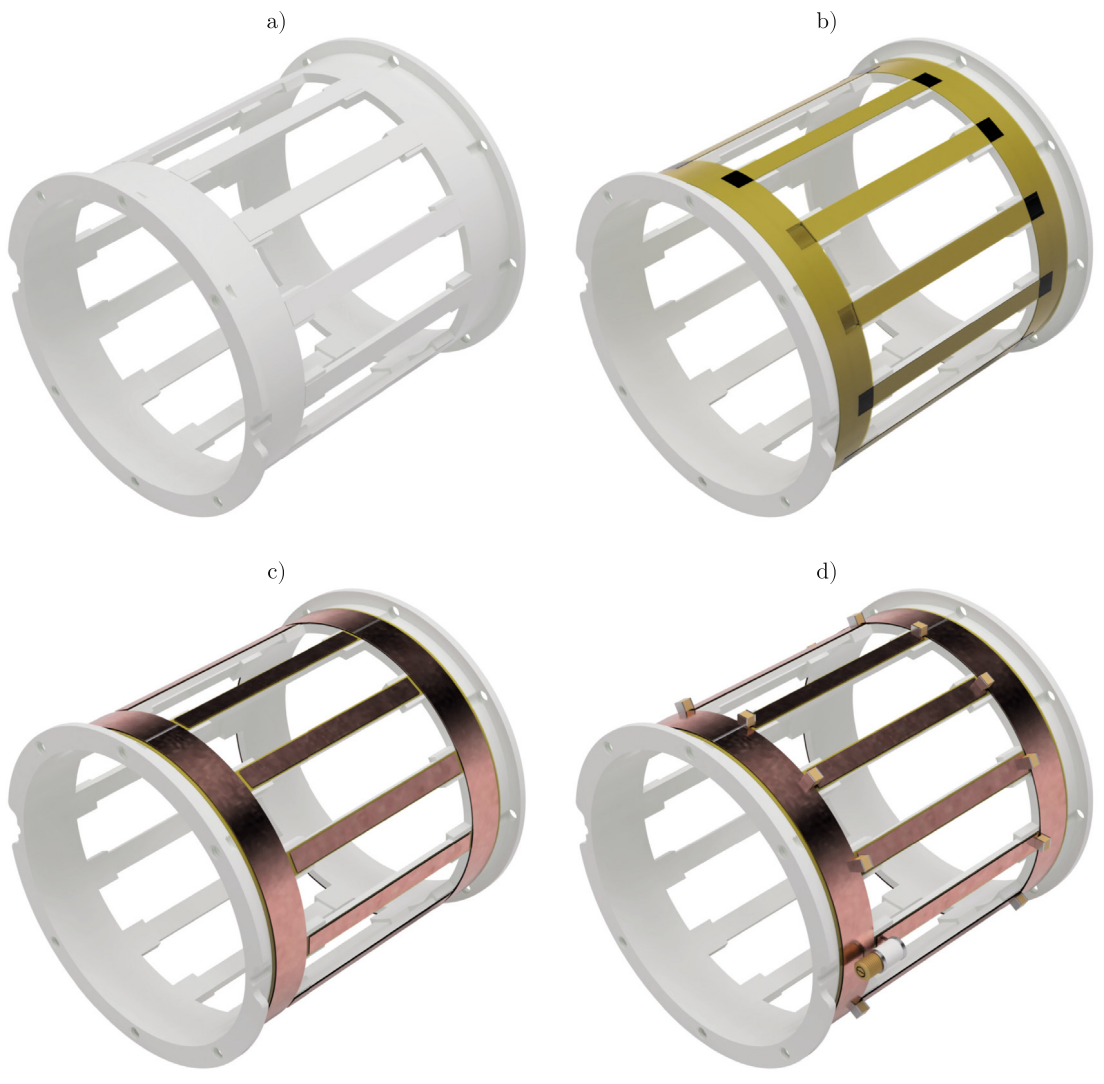
Sequential steps of the BC structure assembly. a) 3D printed structure. b) Application of Kapton tape. c) Application of copper stripes. d) Finished resonant structure with soldered capacitors.

The first step is the assembly of the BC structure from the 3D printed parts (Fig. 3a)). FFF_endring_trimmer, FFF_endring_no_trimmer and twelve times FFF_rung are required.

1. Check the 3D printed parts. If necessary, clean and deburr them.
2. Use an M4 tap to cut threads into all holes of both ER.
3. Place one of the ERs on a flat surface.
4. Add a small drop of superglue to one of the twelve rectangular cavities.
5. Insert one rung and apply constant pressure during curing with a small spring clamp. Pay attention to a tight and straight fit of the rung inside the mount. Remove the clamp after curing.
6. Repeat step 4. and 5. for all remaining rungs. Multiple legs can be glued in at the same time.
7. Tightly fit all twelve legs on the mounts of the second endring.
8. Carefully bend up one of the legs and apply a drop of superglue to the underlying mount.
9. Apply constant pressure during curing with a small spring clamp. Pay attention to a tight and straight fit of the rung inside the mount. Remove the clamp after curing.
10. Repeat step 8. and 9. for all remaining rungs. Not more than four legs should be glued in at the same time.
11. If necessary, clean up excessive glue residues with a sharp knife or sandpaper.
  Prior to soldering, the structure has to be made electrically conductive. The rungs and endrings are cut to size from self-adhesive conductive copper tape (Fig. 3c)). Kapton tape is used in-between the 3D printed PLA parts and the copper tape as a heat barrier for soldering (Fig. 3b)).
12. Take the previously constructed structure and wipe down the top surfaces of the rungs and endrings with isopropanol to remove any dust and grease.
13. Prepare an approximately 70mm long stripe of 6.35mm wide Kapton tape and press it firmly onto one leg, such that it overlaps onto the endrings about the same distance on both sides.
14. Repeat step 13. for all legs.
15. Prepare two approximately 270mm long stripes of 6.35mm wide Kapton tape and place them in parallel with slight overlap around the circumference of the endring, starting at the edge near the legs.
16. Repeat step 15. for the second endring.
17. Use a sharp knife and a ruler to cut out two 262mm x 10mm and twelve 58mm x 5mm strips of self-adhesive conductive copper tape. Maintaining the correct width of the copper tape is crucial; be as precise as possible!
18. Firmly press the long stripe around the circumference of the endring, along the edge towards the legs. Overlapping material at both ends is removed. At best, the seam is located at one of the legs.
19. Repeat step 18. for the second endring.
20. Take one of the prepared short copper stripes and place it onto a rung so that a small gap of the same size is present between the stripe and the ER on both sides. Firmly press the copper tape into place.
21. Repeat step 20. for all legs.
22. The legs must not touch the endrings! Use a multimeter and check that there is no electrical continuity between the legs and the ERs.
  The final step to complete the resonant structure is by soldering the capacitors onto the copper foil (Fig. 3d)). It is important to keep the contact time during soldering as short as possible to minimize the heat input. The PLA tends to deform very quickly under heat. Also, try to keep the amount of solder to a minimum.
23. Solder together the two ends of each ER.
24. Capacitors are placed everywhere between the leg and the rung except for the location where the ER FFF_endring_trimmer has the two cut-outs for the trimmers.
25. Grab one ceramic capacitor (4.7pF, 2kV, Knowles) with a pair of tweezers and position it over a gap between the leg and the ER.
26. Apply a small(!) amount of solder to one contact of the capacitor and the leg to tack it down.
27. Let it cool for a few seconds to avoid warping of the PLA.
28. Tack down the second contact of the capacitor to the ER with a small(!) amount of solder. Applying gentle pressure onto the capacitor with a pair of tweezers can help it to sit flat on the ER.
29. Repeat step 25. to 28. for all the other capacitors (4.7pF, 2kV, Knowles).
30. Bend the legs of the trimmer outwards using pliers such that it can be easily soldered onto the copper foil.
31. Fit FFF_endplate_trimmer to FFF_endring_trimmer with M4 x 8mm screws and check that the trimmer is accessible through the endplate. If it does not fit, adjust the legs.
32. Solder the variable capacitor in place (0.6pF–4.0pF, Knowles).
33. Repeat step 30. to 32. for the second trimmer (0.6pF–4.0pF, Knowles).

### 5.2 Assembly of the pickup loop

**Fig. 4:**
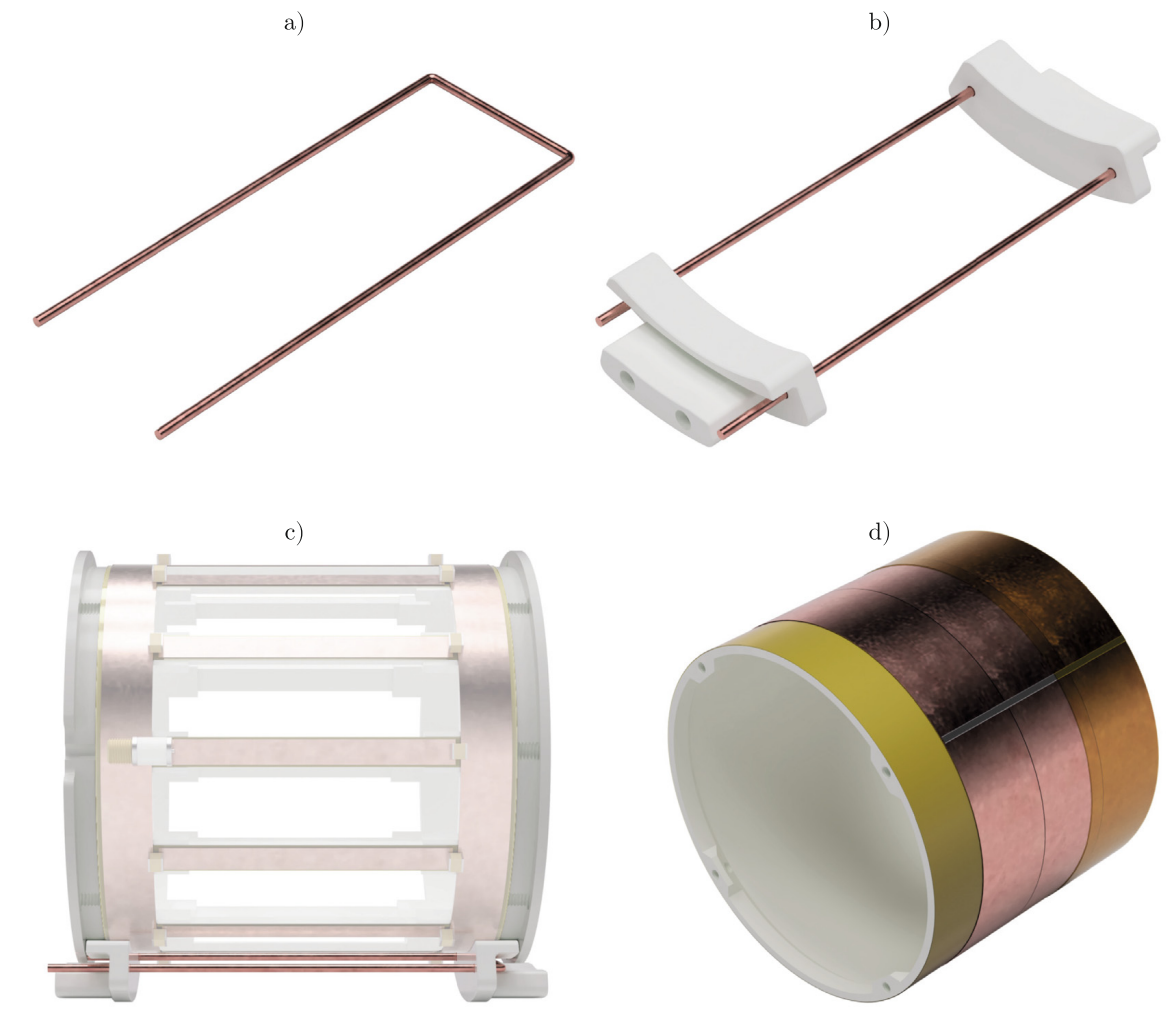
Sequential steps of the pickup loop and shield assembly. a) U-shaped copper wire. b) Copper wire inserted into holders. c) Placement of holders on the circumference of the resonant structure. d) Cutaway view of the shield showing the different layers of Kapton tape and copper foil.

Next, the assembly of the pickup loop is described (Fig. 4a - c). This step requires two times FFF_holder_pickup_loop and FFF_endplate_pickup_loop_trimmer.

1. Check the 3D printed parts. If necessary, clean and deburr them.
2. Use an M4 tap and cut two threads into each holder.
3. Depending on the diameter of the copper wire, the holes in the holders may need to be enlarged with a small drill bit.
4. Strip off the insulation of a 270mm long solid core copper wire ($d_{\mathrm{wire}}$ = 1.7mm) and straighten it by pulling the ends with two pliers.
5. Bend a 34mm wide u-shaped loop with two parallel strands of approximately the same length (Fig. 4a)). Use pliers to bend sharp rectangular corners.
6. Slide both holders over the wire with the tapped holes facing outwards (Fig. 4b)). The wire should sit tightly inside both holders.
7. Take the previously prepared BC structure and place the holders on its circumference (Fig. 4c)). Pull both holders outwards such that they sit flush with both ER and carefully hold them in place with two clamps.
8. Apply a small amount of hot glue between the wire and the holders to keep it from moving. After cooling, the clamps are removed and the basic pickup loop is finished.

### 5.3 Assembly of the shield

The shield only uses the 3D printed part FFF_shield (Fig. 4d)).

1. Check the 3D printed parts. If necessary, clean and deburr them.
2. Use an M4 tap and cut threads into all holes of the shield.
3. Wipe down the surfaces of the printed part with isopropanol to remove any dust and grease.
4. Take the 30mm wide Kapton tape and warp it around the circumference of the shield. Place multiple rings of tape with a slight overlap next to each other to cover the complete shield.
5. Use strips of conductive copper tape to place multiple rings around the shield. The strips are cut to stop and do not overlap at the ends. The individual rings do not overlap either. Firmly press the copper tape onto the cylinder.
6. Solder each copper strip together at the ends to establish electrical conductivity. Further solder joints are placed between the adjoining strips every 45° around the circumference.
7. The copper shielding is covered with a protective layer of Kapton tape. Use the same technique as in step 4.

### 5.4 Assembly of the resonator

**Fig. 5:**
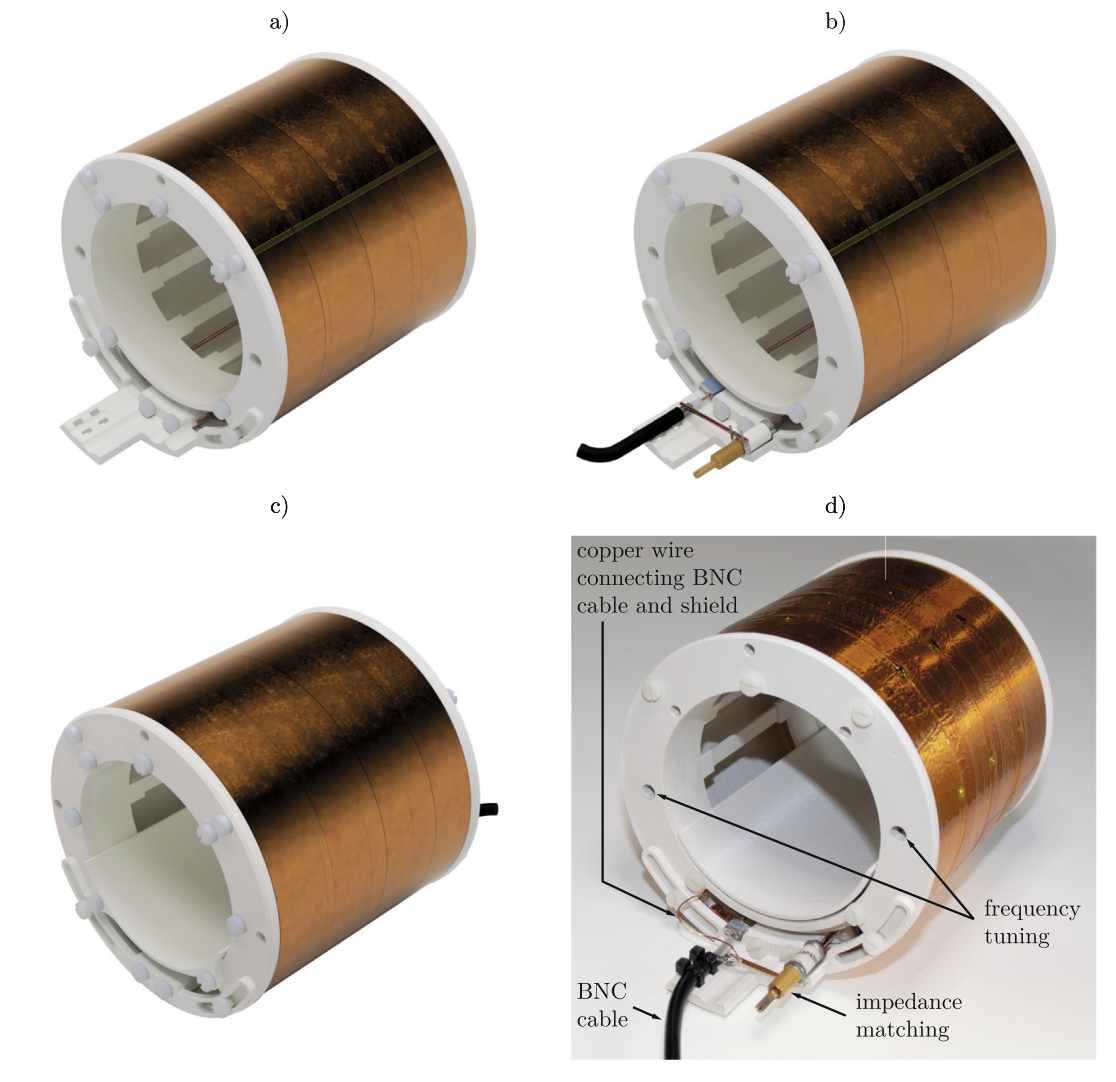
Sequential steps of the final BC assembly. a) Assembly of the prepared parts. b) Capacitors and cable soldered to the pickup loop. c) Installation of the sample tray from the back side. d) Picture of the finished BC resonator.

The last step requires the previously prepared parts as well as the remaining 3D printed components (Fig. 5).

1. Check the 3D printed parts. If necessary, clean and deburr them.
2. Take the part FFF_endplate_trimmer and screw it onto BC structure (Section 5.1) with five M4 x 8mm screws. Check for correct alignment of the trimmers and the access holes.
3. Position the pickup loop (Section 5.2) on the circumference of the BC structure, such that the copper wires are sticking through the opening of the endplate.
4. Hold the pickup loop in position and place the shield (Section 5.3) around the assembly. Use two M4 x 8mm screws located away from the opening to connect the shield to the endplate.
5. FFF_endplate_pickup_loop_trimmer is bolted to the bracket of the pickup loop using two M4 x 8mm screws. Two M4 x 10mm screws are fitted to the slotted holes and are loosely tightened. They serve as a guide and give the loop some freedom of movement around the perimeter.
6. Secure the endplate FFF_endplate_no_trimmer on the other side of the resonator using five M4 x 8mm screws. Pay attention to the correct positioning of the opening such that the bracket of the pickup loop is passed through.
7. FFF_endplate_pickup_loop_no_trimmer is mounted to the other side of the pickup loop as in step 5.
8. Check that the pickup loop is movable between the loop and shield with little force (Fig. 5a)). Adjust the screws through the slotted holes accordingly.
9. Cut the wires of the u-shaped pickup loop to length and solder both capacitors (trimmer: 1.5pF–40pF, Knowles and ceramic capacitor: 4.7pF, 3.6kV, Knowles) as close to the resonator as possible. FFF_endplate_pickup_loop_trimmer can be removed for easier access while soldering.
10. Strip off the insulation of the BNC cable and solder the conductor to the ceramic capacitor (4.7pF, 3.6kV, Knowles).
11. Solder a straight piece of copper wire between the trimmer (1.5pF–40pF, Knowles) and the BNC cable’s shield (Fig. 5b)).
12. Two cable ties are used as a strain relief (Fig. 5d)).
13. Solder a 50mm–60mm long enameled copper wire from the shield to the shielding of the BNC cable(Fig. 5d)). The wire should be routed through the opening of the endplate and soldered to the shield. A small amount of Kapton tape has to be removed from the shield in order to solder to the copper layer.
14. Use two M4 x 10mm screws to install the FFF_sample_tray directly over the pickup loop (Fig. 5c)).

## 6 Operation instructions

When working on MR scanners, there is an increased risk of accidents from the strong static magnetic field of the system which is permanently present. Before working the first time on an MR scanner, instruction by qualified staff is mandatory. It is of uttermost importance that no ferromagnetic objects (screws, tools, watches, smartphones, ...) are brought near the scanner, as they are accelerated by the strong magnetic field acting as projectiles. Persons wearing pacemakers or medical implants are strictly prohibited from working on the scanner! The BC resonator does not pose an increased risk of accidents as only non-ferromagnetic materials were used in the design.

The proposed resonator is used as any other MRI specific coil. The BNC cable is connected to the transmit/ receive switch of the MRI hardware. The resonator is centered inside the MR magnet and the sample is placed on the resonator’s tray. Tuning and matching of the coil has to be performed for optimal SNR and image contrast. Frequency is adjusted by the two trimmers, accessible through the endplate (see Fig. 5d)). It is advisable to fully turn up/ down both trimmers as a starting point and change them both by roughly the same amount to maintain symmetry of the electromagnetic field inside the resonator. Fine-tuning can be done with only one trimmer until the desired frequency is set. Impedance matching uses only a single trimmer attached to the pickup loop (see Fig. 5d)). It is adjusted until the amount of reflected power is minimized. Since tune and match parameters influence each other, the process has to be repeated until resonance frequency and reflected power are optimized. Afterwards, the system is shimmed and images are acquired using the specific MRI software (ParaVision 6.0.1).

## 7 Validation and characterization

To assess the performance of the resonator, three different scenarios were tested. First, the image quality over the entire volume was examined with a bottle of rapeseed oil (Section 7.1). In a second experiment, a water filled ping-pong ball was placed inside the resonator to simulate a higher coil loading (Section 7.2). A lime was used as a phantom for a third qualitative measurement series (Section 7.3). The image quality of the proposed design and a commercial coil was qualitatively compared.

### 7.1 Assessing the volumetric performance with a bottle of rapeseed oil

**Fig. 6:**
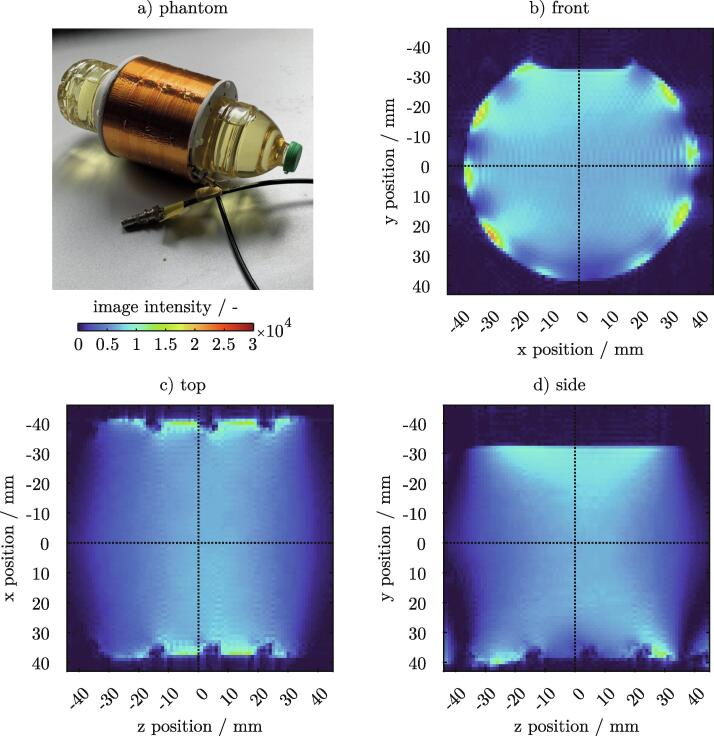
A bottle of rapeseed oil a) is used as a phantom for volumetric FLASH measurements. $T_{1}$-weighted intensity images of all three center planes are shown in b - d). 3D FLASH sequence parameters: FOV  = 128 x 90 x90mm, RES  = 1.0 x 1.0 x 1.0mm, TE  = 4.0ms, TR  = 50ms.

Construction errors or design flaws can be easily detected with a 3D measurement. Initial tests of the resonator were performed with a large volumetric sample, filling the entire space inside the BC (Fig. 6a)). For this, a 1l bottle of rapeseed oil was used. Oil resulted in a moderate loading of the coil which helped to avoid image artefacts at the phase boundary. Multi-slice 3D images were acquired using a FLASH sequence with isotropic resolution of 1.0mm. The $T_{1}$-weighted intensity images of all three center planes are shown in Fig. 6b - d). Intensity fluctuations of the $T_{1}$ signal were visible in all three views, especially in the vicinity of the rungs. Additionally, an increase in signal intensity was observed near the oil surface (Fig. 6b, d)). This was caused by susceptibility changes near the phase boundary. The signal intensity increased in the surrounding of the capacitors. This effect was most visible on the resonator’s bottom where the rung is cut by the side view’s center plane (Fig. 6d)). At $z_{\mathrm{pos}}$ = -30mm and $z_{\mathrm{pos}}$ = +30mm the leg and endrings were connected to each other by capacitors. The notches with defined spacing along the z-axis (Fig. 6b, d)) were caused by a direct replication of the specific bottle geometry (see Fig. 6a)).

**Fig. 7:**
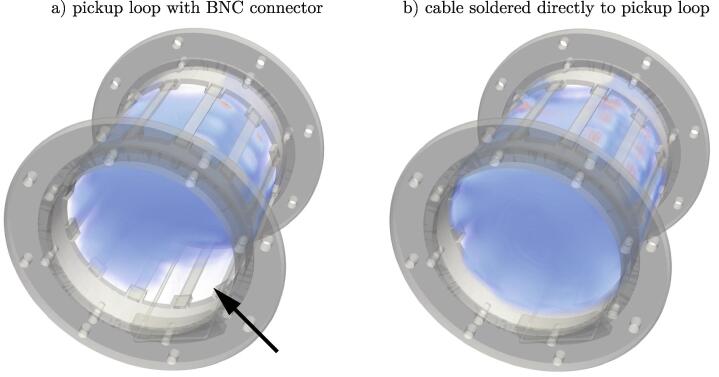
Comparison of the same resonant birdcage structure using a pickup loop with a static BNC connector a) and a directly soldered cable without connector b). The measured volumetric FLASH data and the CAD design of the resonator are superimposed for better visualization. The presence of a BNC connector directly influences the field inside the BC and results in a signal loss in its close environment, showing that design choices have to be carefully considered.

The effect of an unfavorable design decision is shown in Fig. 7. In an earlier version a BNC connector was soldered directly to the pickup loop for an ease of handling (Fig. 7a)). The presence of the connector had a major impact on the uniformity of the electromagnetic field. Due to the slight ferromagnetic properties of the connector, the field in its vicinity was disturbed to such an extent that the signal was fully eliminated (see arrow Fig. 7a)). Even repeated shimming could neither eliminate nor reduce this flaw. The BNC cable was soldered directly to the pickup loop for a permanent fix of the issue (see Fig.7b)).

**Fig. 8:**
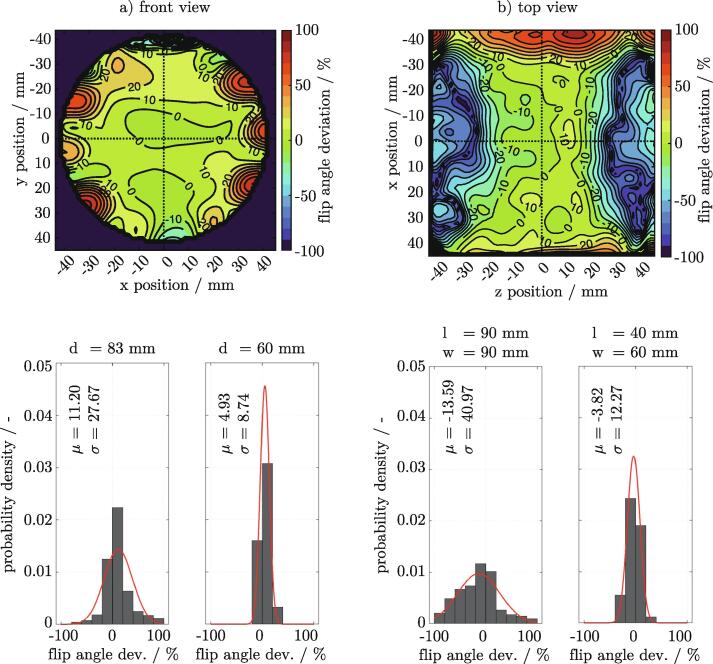
Uniformity of the resonators electromagnetic field within the front a) and top b) center plane. The double angle method [15] is used for $B_{1}$ mapping. Field homogeneity is expressed as the percentage deviation of a reference flip angle of $\alpha_{1}$ = 30°. The influence of the FOV size on the distribution of the flip angle deviation is given by the probability density plots. The left distribution in each plot shows the flip angle deviation distribution for the full shown FOV, whereas the right plot indicates a narrower distribution at a reduced FOV. 2D FLASH sequence parameters: FOV_front_ = 90 x 90mm, FOV_top_ = 180 x 90mm, RES  = 1.406 x 1.406mm, SLTH  = 2.0mm, TE  = 4.0ms, TR  = 6000ms, FA($\alpha_{1}$) = 30°, FA($\alpha_{2}$.) = 60°.

In quantitative imaging, irregular $B_{1}$ field distributions inside the resonator represent a significant source of error. Looking at $T_{1}$ contrast measurements, for example, this may result in intensity fluctuations across the entire FOV. $B_{1}$ mapping is often used to correct this error subsequently. The $B_{1}$ map gives a direct overview of where local variations of the field occur, thus making it suitable for quantification of the BC’s field uniformity. The $B_{1}$ field of the BC was mapped in multiple axial and transversal slices using the simple double angle method [15]. Two $T_{1}$ intensity images with flip angles of $\alpha_{1}$ = 30° and $\alpha_{2}$ = 60° were acquired with all other parameters left unchanged. Assuming that complete recovery over TR and no $T_{1}$ dependency is given, the flip angle distribution can be calculated. Fig. 8 shows the field homogeneity in the front and top center plane, expressed as the percentage deviation of the reference flip angle $\alpha_{1}$ = 30°.

A $d_{\mathrm{mask}}$ = 83mm circular mask was applied to the data of the front center plane (Fig. 8a)), which matches the diameter of the resonant birdcage structure. The field distribution in the center of the plane proved to be uniform with minor local deviations of up to ± 5%. The variations of the field increased towards the edge of the masked region with hotspots formed in the vicinity of the rungs. This behavior is observed in many BC designs and in good agreement. The hotspots are explained by the currents circulating in the rungs which add to the generated magnetic field. This contribution is locally limited and declines with increasing distance towards the resonators center. A comparison of the normally distributed probability density distributions (Fig. 8a)) showed that a decrease in mask diameter from $d_{\mathrm{mask}}$ = 83mm to $d_{\mathrm{mask}}$ = 60mm reduced the standard deviation σ by a factor of almost 3.2. Thus, it is best to select a sample size that does not occupy the entire volume of the resonator.

A similar influence of the legs on the magnetic field was also observed from the top view (Fig. 8b)). A quadratic mask of $l_{\mathrm{mask}}$ = 90mm and $w_{\mathrm{mask}}$ = 90mm was applied to the top center plane. Field variations increased strongly in the direction of the rungs ($x_{\mathrm{pos}}$ > +30mm and $x_{\mathrm{pos}}$ < -30mm). Different from the front view, a field gradient in z direction with a maximum intensity in the middle of the resonator ($z_{\mathrm{pos}}$ = 0mm) was observed. This declining intensity from the center towards the ends can be observed in all simple BC designs and is in good agreement. The steepness of the gradient is significantly influenced by the length of the BC. A longer resonator would therefore offer a larger, more homogeneous measuring range. The normally distributed probability density distributions (Fig. 8b)) clearly indicated that a narrower mask of $l_{\mathrm{mask}}$ = 40mm and $w_{\mathrm{mask}}$ = 60mm lowered the standard deviation σ by a factor of approximately 3.3. For this reason, samples should be kept short for best results.

### 7.2 Testing a higher coil loading with a water filled ping-pong ball

**Fig. 9:**
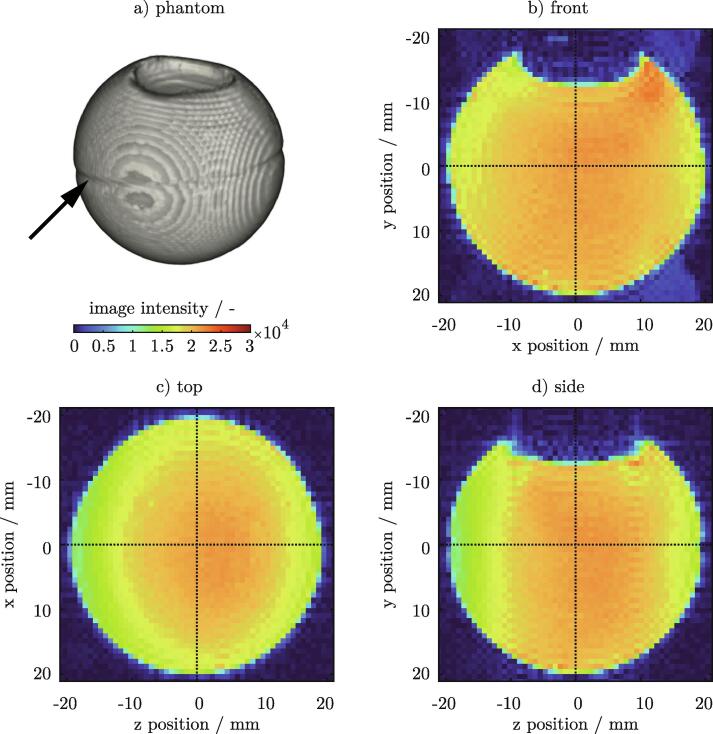
Volumetric FLASH measurements using a water filled ping-pong ball a) as a small spherical phantom ($d_{\mathrm{ball}}$ = 40mm). $T_{1}$-weighted intensity images of all three center planes are shown in b - d). The phase boundary between the water and the entrapped air inside the phantom is visible towards the top of the front b) and side d) view. The volumetric view a) reveals a seam (see arrow) from the manufacturing process of the ball running around its circumference. 3D FLASH sequence parameters: FOV  = 50 x 50 x 50mm, RES  = 0.781 x 0.781 x 0.781mm, TE  = 2.0ms, TR  = 100ms.

A second experiment was performed to test the influence of a higher coil loading on the image quality. For this, a water filled ping-pong ball ($d_{\mathrm{ball}}$ = 40mm) was used as a phantom. The ball was small enough to fit well into the section of the resonator where the electromagnetic field was most uniform (Section 7.1). A 3D FLASH measurement with an isotropic resolution (RES  = 0.781 x 0.781 x 0.781mm) was performed. The volumetric representation revealed a seam running around the circumference of the ball (see arrow Fig. 9a)) where the two plastic halves were fused together during production. The dent on top was caused by a small amount of air entrapped inside the phantom. This was particularly suitable for a qualitative observation, since strong differences in susceptibility occur at gas/ liquid phase boundaries and thus, often lead to image artifacts. $T_{1}$-weighted intensity images of all three center planes are presented in Fig. 9b - d). No distortions were visible in any of the views. However, the front and the side view indicated a small increase in signal intensity around the top phase boundary due to the change in susceptibility.

### 7.3 Performing qualitative measurements with a lime as a phantom

**Fig. 10:**
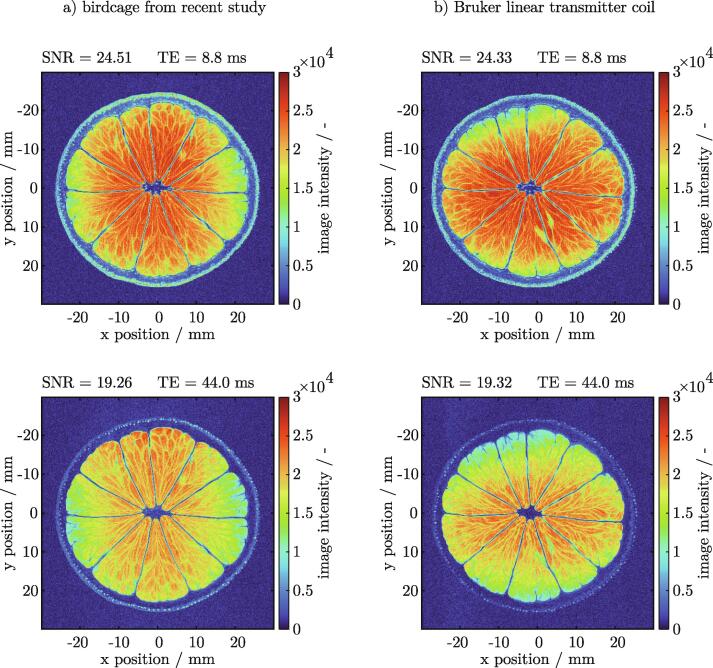
Qualitative comparison between the proposed design a) and a Bruker Linear Transmitter Coil (Part No T10720V3) b) using a lime as a phantom. 2D RARE images are taken with two different echo times TE  = 8.8ms and TE  = 44.0ms. For both resonators, the structure of the fruit is well resolved. The SNR is calculated from the ROI (lime) and the noise level of the background [19]. and can be regarded as equal between both setups. 2D RARE sequence parameters: FOV  = 50 x 50mm, RES  = 0.117 x 0.117mm, SLTH  = 3.0mm, TE  = 8.8ms, TR  = 7500ms, RARE Factor  = 2, .Averages  = 2.

A lime was used as a specimen to assess the qualitative performance of the proposed design. RARE images of 2D slices were acquired with two different echo times TE  = 8.8ms and TE  = 44.0ms. Well resolved images were obtained with an in-plane resolution of RES  = 0.117 x0.117mm and a slice thickness of SLTH  = 3.0mm. The same measurement was repeated using a commercial linear birdcage resonator (Bruker Linear Transmitter Coil, Part No T10720V3). A side-by-side comparison of the images is given in Fig. 10. Minor structural differences were visible between the two series of measurements, since an exact repositioning of the lime was not realizable.

The SNR was determined from the mean intensity of th ROI (lime) and the noise level of the background according to Dietrich et al. [19]. The calculated SNR can be regarded as equal between both setups. An overall higher signal intensity was observed for the measurement with short TE. The epidermis [20] and the region of the peel in between the skin and the segments was more pronounced. The juice vesicles within the individual segments were clearly visible at both echo times. A minor horizontal intensity gradient was noticeable inside the image taken with the proposed BC design. Likewise, a measurement with the Bruker BC revealed a very similar picture with a slight intensity gradient, however in a different direction most likely due to the placement of the pickup loop inside the resonator. Unfortunately, because of warranty reasons, the Bruker resonator could not be opened to examine its internal design. Both BC resonators performed equally well for this application.

## 8 Summary

This work reports on the design and construction of an inductively coupled linear birdcage resonator suitable for magnetic resonance imaging. The aim was to develop an open-source 3D printable resonator which can be built on a low budget, even by non-professionals in the field of MRI coil design. The specific design decisions were outlined for better understanding and as a guide to adapt the setup to other use cases. The experiments showed that the proposed design is capable of creating well resolved images using a variety of different phantoms. It was also confirmed that the $B_{1}$ field inside the resonator is mostly uniform for small samples of $d_{\mathrm{sample}}$ = 60mm and $l_{\mathrm{sample}}$ = 40mm. Objects of up to $d_{\mathrm{sample}}$ = 83mm and $l_{\mathrm{sample}}$ = 90mm can be measured with the BC but will suffer from $B_{1}$ inhomogeneities towards the resonant structure and the ends of the BC. The design worked well for the presented use cases. It is intended to implement a flow setup for hydrodynamic investigations of two-phase gas/liquid flows in the next step.

## Declaration of Competing Interest

The authors declare that they have no known competing financial interests or personal relationships that could have appeared to influence the work reported in this paper.
